# Appropriate ICD Therapies in Primary Prevention: A Propensity‐Matched Comparison of Contemporary and Historic Heart Failure Cohorts

**DOI:** 10.1002/joa3.70497

**Published:** 2026-10-08

**Authors:** Inês Ferreira Neves, Guilherme Portugal, Julien Lopes, Ana Rita Teixeira, Pedro Silva Cunha, Bruno Valente, Ana Lousinha, Rita Moreira, Hélder Santos, António Gonçalves, Rui Cruz Ferreira, Mário Martins Oliveira

**Affiliations:** ^1^ Hospital de Santa Marta Unidade Local de Saúde S. José, Centro Hospitalar Universitário de Lisboa Central Lisboa Portugal

**Keywords:** guideline‐directed medical therapy, heart failure, implantable cardioverter defibrillator, primary prevention, sudden cardiac death

## Abstract

**Background:**

In heart failure with reduced ejection fraction (HFrEF), a substantial proportion of deaths are arrhythmic. Implantable cardioverter‐defibrillators (ICDs) are recommended for primary prevention in patients with left ventricular ejection fraction (LVEF) ≤ 35% despite guideline‐directed medical therapy (GDMT), but landmark trials predate widespread use of angiotensin receptor–neprilysin inhibitors (ARNI) and sodium–glucose cotransporter 2 inhibitors (SGLT2i), raising uncertainty about arrhythmic risk in the contemporary treatment era.

**Methods:**

We retrospectively studied consecutive HFrEF patients undergoing primary‐prevention ICD implantation at a single center (2015–2022), categorized into historical‐era and contemporary‐era cohorts by ARNI/SGLT2i adoption. Propensity‐score matching balanced baseline characteristics. The primary endpoint was time to first appropriate ICD therapy (antitachycardia pacing or shock).

**Results:**

Of 289 patients (mean age 62 ± 11 years, 82% male), 234 (117 per group) remained after matching. Mean follow‐up was 51 months. Appropriate ICD therapy incidence did not differ significantly between contemporary‐era and historical‐era cohorts (hazard ratio [HR] 1.52, 95% CI 0.84–2.72; *p* = 0.165). Competing‐risk‐adjusted cumulative incidence was numerically higher in the contemporary‐era cohort at a pre‐specified 36‐months horizon, consistent with a sensitivity analysis using an expanded propensity‐score model (HR 1.55, 95% CI 0.82–2.94).

**Conclusion:**

ICD therapy rates were not significantly different between the contemporary‐era and historical‐era cohorts, a finding robust to sensitivity and competing‐risk analyses. These results should be interpreted cautiously, as the study may be underpowered to detect modest differences, and clinically relevant effects cannot be excluded.

## Graphical abstract

No significant difference in appropriate ICD therapies between treatment eras. Time to first ICD therapy was similar after propensity‐score matching. The result was robust to a sensitivity analysis using an expanded propensity model. Cumulative incidence, accounting for competing risk of death, was also higher. ICD candidates, despite modern GDMT, may represent a higher‐risk subgroup.

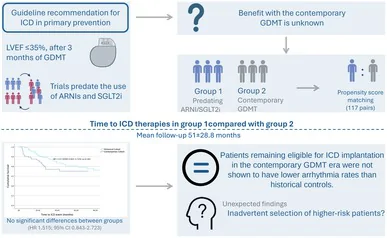

**Abbreviations**
ACEI: angiotensin‐converting enzyme inhibitor
ARB: angiotensin receptor blocker
ARNI: angiotensin receptor‐neprilysin inhibitor
CI: confidence interval
CRT: cardiac resynchronization therapy
GDMT: guideline‐directed medical therapy
HFrEF: heart failure with reduced ejection fraction
HR: hazard ratio
ICD: implantable cardioverter‐defibrillator
ICM: ischemic cardiomyopathy
LVEF: left ventricular ejection fraction
MI: myocardial infarction
NICM: nonischemic cardiomyopathy
NYHA: New York Heart Association
OR: odds ratio
RCT: randomized controlled trial
SCD: sudden cardiac death
SGLT2i: sodium–glucose cotransporter 2 inhibitor
VF: ventricular fibrillation
VT: ventricular tachycardia

## 1 Introduction

Among patients with heart failure with reduced ejection fraction (HFrEF), a significant proportion of deaths occur due to electrical disturbances, including ventricular tachyarrhythmias (VT/VF), bradycardia, and asystole [1]. Implantable cardioverter‐defibrillators (ICD) are effective in reverting VT/VF and are, as such, recommended in primary prevention to reduce sudden cardiac death (SCD) and all‐cause mortality in patients with symptomatic HFrEF with left ventricular ejection fraction (LVEF) ≤ 35%, in patients meeting guideline criteria following optimization of guideline‐directed medical therapy (GDMT) [1]. The implantation of an ICD in these patients is a Class I, level of evidence A, for patients with ischemic etiology, but the evidence is less strong for patients with nonischemic cardiomyopathy (NICM) [1].

In recent years, GDMT for HFrEF has expanded to include angiotensin receptor–neprilysin inhibitors (ARNI) and sodium–glucose cotransporter 2 inhibitors (SGLT2i), alongside beta‐blockers and mineralocorticoid receptor antagonists [1]. The major randomized trials supporting ICD use for primary prevention were conducted before these agents were available. Contemporary evidence, particularly from randomized controlled trials, is lacking [2, 3, 4, 5, 6, 7]. Our aim was to evaluate whether patients under GDMT for HFrEF, including ARNI and SGLT2i inhibitors, experience similar rates of arrhythmic events requiring ICD therapy following primary prevention ICD implantation.

## 2 Methods

### 2.1 Study Population

This retrospective study enrolled consecutive adult patients with HFrEF, with either ischemic or nonischemic etiology, symptomatic NYHA Class II‐III, and with LVEF ≤ 35% after 3 months of GDMT, who were implanted with an ICD for primary prevention at our center between January 2015 and December 2022. Indication for implantation was based on published international guidelines [1]. Appropriate ICD therapy was defined as the first antitachycardia pacing (ATP) or shock delivered for documented ventricular tachycardia (VT) or ventricular fibrillation (VF). All patients were referred for ICD implantation according to guideline‐based criteria following clinical optimization of GDMT.

Device programming at implantation was identical for all the included patients and consisted of VT zone of 171–204 bpm, with ATP and subsequent shock, and a VF zone above 205 bpm.

All events were individually reviewed by an external observer.

Patients with NYHA Class IV, coronary revascularization in the previous 3 months, myocardial infarction (MI) in the previous month or with documented VT/VF or SCD were not included. Patients undergoing concomitant cardiac resynchronization therapy (CRT) were excluded to avoid confounding effects on outcomes.

Cohorts were defined using a temporal cutoff of June 30, 2018, selected to reflect the transition to routine clinical use of ARNI. In our healthcare setting, ARNI became co‐financed by the national healthcare system in November 2017; a 6‐month interval was therefore allowed to enable treatment uptake during follow‐up heart failure consultations and to better capture changes in prescribing patterns. This era‐based classification was used as a pragmatic proxy for treatment exposure. Throughout this manuscript, the two groups are referred to as the historical‐era and contemporary‐era cohorts to reflect the timing of ICD implantation, rather than uniform exposure to contemporary GDMT; as detailed below, medication use varied within both cohorts.

The study followed the principles outlined in the Declaration of Helsinki. The institutional ethics committee approved the study protocol.

### 2.2 Outcomes

The primary endpoint was time to first appropriate ICD therapy (ATP or shock).

Secondary endpoints were time to first ICD shock and time to first administered ATP therapy.

### 2.3 Statistical Analysis

Statistical analysis was performed using the Statistical Package for the Social Sciences, version 24.0 for Windows (SPSS). Point estimates and 95% confidence intervals (CIs) are described for all mean estimates. Descriptive variables were summarized with the use of frequency distributions, means and standard deviations, or medians and interquartile ranges and were tested with the use of Fisher's exact test, *t*‐tests, or the Wilcoxon–Mann–Whitney test, as appropriate.

To address potential confounding due to the nonrandomized nature of the study, propensity‐score matching (PSM) was performed to balance baseline characteristics between the historical‐era and contemporary‐era cohorts. A logistic regression model was used to estimate the probability of a patient being included in the contemporary‐era cohort based on predefined baseline variables: age, etiology of heart failure, LVEF before ICD implantation, QRS duration ≥ 120 ms, and NYHA functional class. Patients from each cohort were matched 1:1 based on their propensity scores using a caliper of 0.005. Covariate balance after matching was assessed using standardized mean differences (SMD), with SMD < 0.10 considered indicative of adequate balance, displayed as a Love plot (Figure S2); this approach replaced reliance on between‐group *p*‐values alone, which are sensitive to sample size and do not quantify the magnitude of residual imbalance. As a sensitivity analysis, propensity‐score estimation and matching were repeated using an expanded model additionally including sex, hypertension, diabetes, dyslipidemia, chronic kidney disease, chronic obstructive pulmonary disease, and amiodarone use; prior myocardial infarction and prior percutaneous coronary intervention were not included in this expanded model because they were quasi‐collinear with ischemic/nonischemic etiology in our cohort. This sensitivity analysis used 1:1 nearest‐neighbor matching without replacement on the logit of the propensity score, with a caliper of 0.2 standard deviations of the logit (propensity score) (Table S1).

ICD therapies (antitachycardia pacing and shocks) were recorded during follow‐up. We analyzed time to first appropriate ICD therapy, time to ICD shock, and time to ATP therapy in the historical‐era (Group 1) and contemporary‐era (Group 2) cohorts using Cox proportional hazards regression and Kaplan–Meier estimation. The proportional hazards assumption was assessed using a Schoenfeld residual‐based test. Given the borderline result obtained, restricted mean survival time (RMST) at a fixed 36‐month horizon was additionally calculated. Because the historical‐era cohort had greater potential follow‐up, follow‐up duration was estimated separately for each cohort using the reverse Kaplan–Meier method. Death was treated as a competing risk for appropriate ICD therapy: the primary Cox model censored patients at their date of death (cause‐specific hazard), and cumulative incidence functions accounting for the competing risk of death were additionally estimated using the Aalen‐Johansen estimator. Effect modification by baseline subgroup was assessed using formal treatment‐era × subgroup interaction terms within Cox model fitted to the full (unmatched) cohort. We considered *p* < 0.05 to indicate a statistically significant difference.

## 3 Results

A total of 289 patients were included (82.4% male, mean age 62.0 ± 11.3 years, IQR 55–70). One hundred forty‐eight patients (51.2%) were in the historical‐era cohort (before ARNI/SGLT2i use), while 141 (48.8%) were in the contemporary‐era cohort. There were no statistically significant differences between groups regarding age, sex, heart failure etiology, LVEF at the time of device implantation, NYHA class, or presence of comorbidities; QRS duration was longer in the contemporary‐era cohort (*p* = 0.012).

Regarding HFrEF medication, 138 (93.2%) of the historical‐era cohort and 51 (36.2%) of the contemporary‐era cohort were medicated with an ACEI or ARB (*p* < 0.001). ARNI use was 0.7% in the historical‐era group versus 59.6% in the contemporary‐era group (*p* < 0.001). SGLT2i use was 2.0% versus 31.9%, respectively (*p* < 0.001).

### 3.1 Propensity Score–Matched Cohort

After propensity‐score matching, 234 patients remained (117 per group). Standardized mean differences (SMD) before and after matching are shown in a Love plot (Figure S2); matching achieved adequate balance (SMD < 0.10) for age, sex, and NYHA class, but several covariates not included in the propensity‐score model remained imbalanced afterward, most notably hypertension (SMD 0.32), prior percutaneous coronary intervention (SMD 0.40), diabetes (SMD 0.27), LVEF (SMD 0.26), and dyslipidemia (SMD 0.26); medication variables that define treatment era (ACEI/ARB, ARNI, SGLT2i) remained markedly different between groups (SMD > 0.85), as expected. In a pre‐specified sensitivity analysis using an expanded propensity‐score model incorporating these additional covariates, nearest‐neighbor matching yielded 97 matched pairs (194 patients) with improved balance for the targeted covariates (Table S2, Figure S2). Pooled mean follow‐up was 51 ± 28.8 years; using the reverse Kaplan–Meier method, median follow‐up was 77.6 months (95% CI 72.6–82.5) in the historical‐era cohort versus 36.2 months (95% CI 31.7–43.8) in the contemporary‐era cohort. Baseline characteristics after propensity‐score matching are presented in Table 1.

**TABLE 1:** Baseline characteristics of the study population and comparison between groups after propensity‐score matching.

	All (*n* = 234)	Group 1 (*n* = 117)	Group 2 (*n* = 117)	*p*
Age (years)—mean ± SD	61.6 ± 11.3	61.5 ± 12.0	61.6 ± 10.6	0.977
Male sex—*n* (%)	190 (81.2)	95 (81.2)	95 (81.2)	1.00
Heart failure etiology				0.892
Ischemic	150 (64.1)	76 (65.0)	74 (63.2)	
Nonischemic	84 (35.9)	41 (35.0)	43 (36.8)	
LVEF (%)—mean ± SD	28.0 ± 5.2	28.8 ± 4.7	27.2 ± 5.5	0.019
QRS duration ≥ 120 ms—*n* (%)	63 (26.9)	30 (25.6)	33 (28.2)	0.768
NYHA class—*n* (%)				0.403
≤ II	190 (81.2)	98 (83.8)	92 (78.6)	
≥ III	44 (18.8)	19 (16.2)	25 (21.4)	
Comorbidities—*n* (%)				
Hypertension	164 (70.1)	90 (76.9)	74 (63.2)	0.035
Type 2 DM	79 (33.8)	32 (27.4)	47 (40.2)	0.077
Dyslipidemia	165 (70.5)	89 (76.1)	76 (65.0)	0.086
Current alcohol abuse	19 (8.1)	13 (11.1)	6 (5.1)	0.144
Previous alcohol abuse	13 (5.6)	6 (5.1)	7 (6.0)	0.584
Smoker	71 (30.3)	35 (29.9)	36 (30.8)	0.602
Previous smoking	56 (23.9)	24 (20.5)	32 (27.4)	0.298
Current drug abuse	4 (1.7)	3 (2.6)	1 (0.9)	0.361
Previous drug abuse	2 (0.9)	1 (0.9)	1 (0.9)	1.000
Chronic kidney disease	41 (17.5)	19 (16.2)	22 (18.8)	0.731
COPD	27 (11.5)	10 (8.5)	17 (14.5)	0.219
Medication—*n* (%)				
ACEI/ARB	153 (65.4)	109 (93.2)	44 (37.6)	< 0.001
ARNI	68 (29.1)	1 (0.9)	67 (57.3)	< 0.001
Beta‐blocker	225 (96.2)	111 (94.9)	114 (97.4)	0.291
Spironolactone	178 (76.1)	84 (71.8)	94 (80.3)	0.106
Dapagliflozin/empagliflozin	31 (13.2)	0 (0.0)	31 (26.5)	< 0.001
Amiodarone	12 (5.2)	6 (5.1)	6 (5.1)	1.000
Ivabradine	33 (14.1)	14 (12.0)	19 (16.2)	0.151
Loop diuretic	154 (65.8)	77 (65.8)	77 (65.8)	0.889

### 3.2 Primary Endpoint

Regarding the primary outcome, no statistically significant difference was observed in time to first appropriate ICD therapy (HR 1.515; 95% CI 0.843–2.723, *p* = 0.165), with a nonsignificant trend toward a higher risk of ICD therapy in the contemporary‐era group (Figure 1A). A Schoenfeld residual‐based test showed a borderline violation of the proportional hazards assumption for treatment era (*p* = 0.050), consistent with visual divergence of the Kaplan–Meier curves that appeared to attenuate over longer follow‐up (Figure 1). At a common fixed 36‐month horizon, cumulative incidence of appropriate ICD therapy was 14.6% (95% CI 8.0%–21.3%) in the historical‐era cohort versus 23.0% (95% CI 14.5%–31.4%) in the contemporary‐era cohort, an absolute risk difference of 8.4% (95% CI –2.4–19.1); restricted mean event‐free survival time at 36 months was 33.7 versus 31.0 months, respectively. Because death precluded subsequent ICD therapy in a substantial proportion of patients (43/117 [36.8%] historical‐era versus 23/117 [19.7%] contemporary‐era), we additionally estimated the cumulative incidence of ICD therapy accounting for death as a competing risk using the Aalen‐Johansen estimator (Figure 1B); competing‐risk‐adjusted estimates were similar to naive Kaplan–Meier estimates at 36 months (14.0% vs. 21.5%) and diverged modestly over full follow‐up (21.6% vs. 23.2%), reflecting greater cumulative mortality in the historical‐era cohort. In the sensitivity analysis using the expanded propensity‐score model (97 pairs, 194 patients), the primary result was materially unchanged (HR 1.55; 95% CI 0.82–2.94; *p* = 0.178).

**FIGURE 1:**
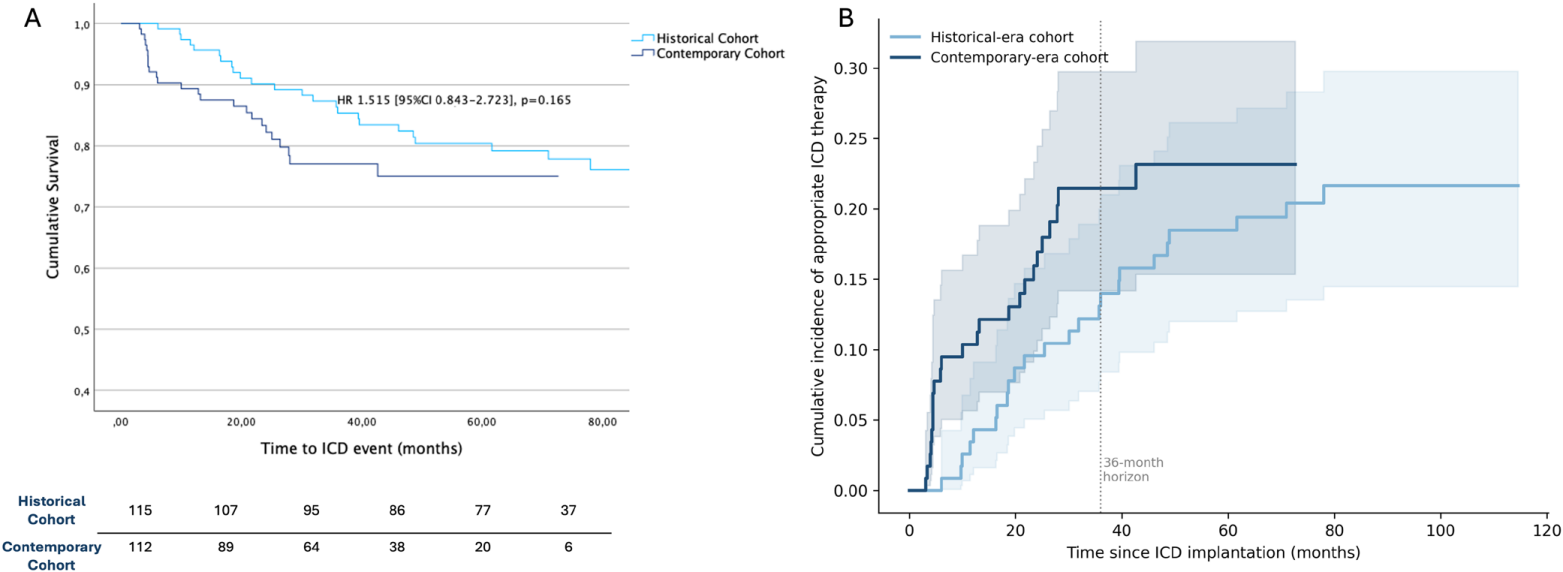
Kaplan–Meier freedom from the primary composite endpoint (Panel A; appropriate ICD therapy, defined as antitachycardia pacing or shock) in the propensity‐score‐matched cohort, with number of patients at risk. (B) Aalen‐Johansen cumulative incidence of appropriate ICD therapy in the same cohort, accounting for death as a competing risk; dotted line indicates the prespecified 36‐month horizon.

### 3.3 Secondary Endpoints

Secondary endpoints included time to first ICD shock and time to first ATP therapy. ICD shocks occurred in 17 patients in the contemporary‐era cohort and 17 patients in the historical‐era cohort (HR 1.90, 95% CI 0.906–3.985, *p* = 0.090). ATP therapies occurred in 19 patients in the contemporary‐era cohort and 21 patients in the historical‐era cohort (HR 1.447, 95% CI 0.751–2.786, *p* = 0.269). Kaplan–Meier curves for the primary and secondary endpoints, with number of patients‐at‐risk tables and event counts, are shown in Figures 2 and 3.

**FIGURE 2:**
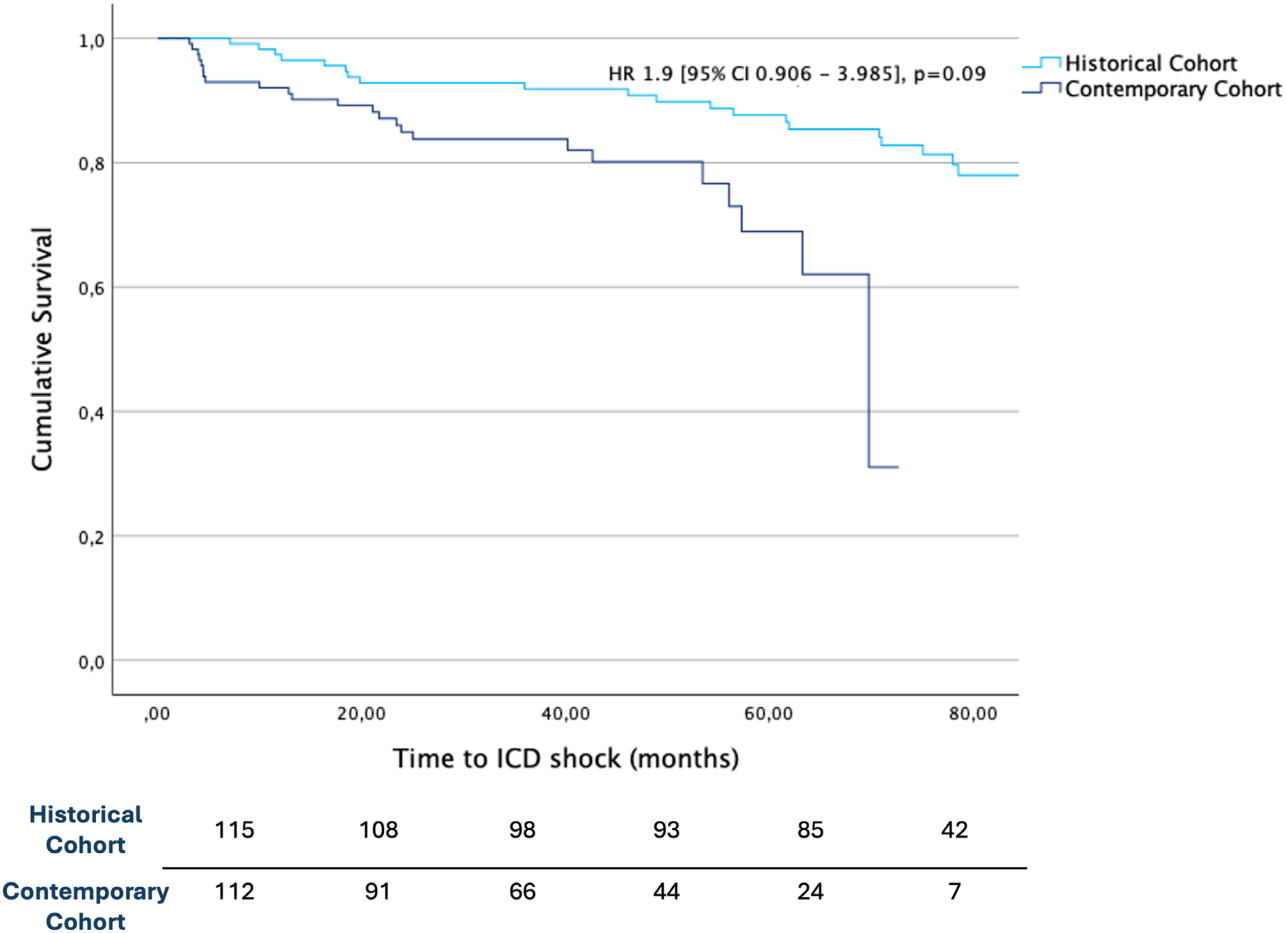
Kaplan–Meier freedom from ICD shock (secondary endpoint) in the propensity‐score‐matched cohort, with the number of patients at risk.

**FIGURE 3:**
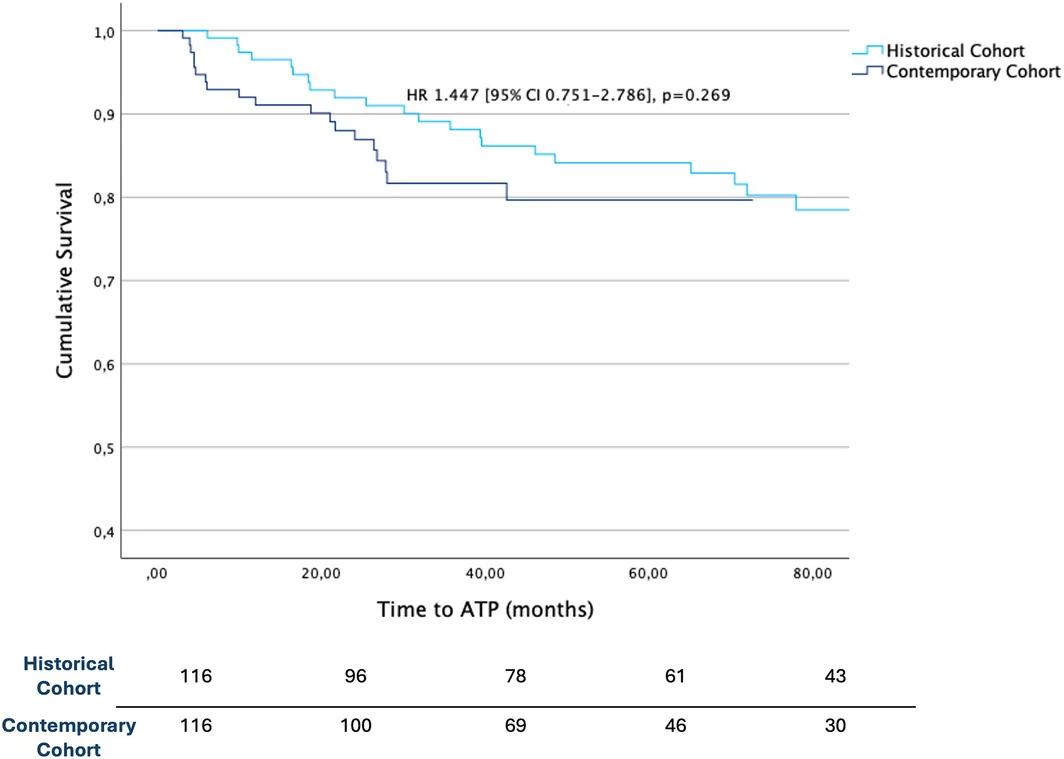
Kaplan–Meier freedom from ATP therapy (secondary endpoint) in the propensity‐score‐matched cohort, with the number of patients at risk.

### 3.4 Subgroup Analysis

In patients with ischemic cardiomyopathy (*n* = 76 historical‐era; *n* = 74 contemporary‐era), the incidence of appropriate ICD therapy was 16/76 in the historical‐era cohort and 15/74 in the contemporary‐era cohort (HR 1.40, 95% CI 0.63–3.12, *p* = 0.412). In nonischemic cardiomyopathy (*n* = 41 historical‐era; *n* = 43 contemporary‐era), the incidence was 8/41 in the historical‐era cohort and 10/43 in the contemporary‐era cohort (HR 1.66, 95% CI 0.75–3.69, *p* = 0.217).

A further exploratory subgroup analysis was performed in the full unmatched cohort to assess whether baseline characteristics modified the association between treatment era and the primary endpoint (Figure S1). No significant interaction was observed for NYHA class (*p* = 0.58), LVEF (*p* = 0.74), or etiology (*p* = 0.38). QRS duration > 110 ms, previously highlighted based on a subgroup‐specific *p*‐value, showed no evidence of interaction when tested formally (interaction *p* = 0.99). Given the limited number of events, all subgroup findings should be considered exploratory and hypothesis‐generating.

## 4 Discussion

In summary, our key findings are:

1. Baseline characteristics of patients undergoing primary‐prevention ICD implantation were largely, though not completely, balanced across eras after propensity‐score matching, with residual imbalance persisting for a subset of covariates.
2. Contemporary ICD recipients more frequently received ARNI and SGLT2i, reflecting broad adoption of current GDMT.
3. We did not observe a statistically significant difference in ICD therapies between the historical‐era and contemporary‐era cohorts over long‐term follow‐up.

ICDs have been proven effective for primary prevention of SCD in HFrEF in multiple randomized trials, including MADIT I, MADIT II, and SCD‐HeFT, [2, 3, 4, 5, 6, 7, 8] though these studies were conducted before ARNI and SGLT2i became standard therapy. More recent trials such as DANISH showed a reduction in SCD in ICD patients but not in all‐cause mortality [9].

Emerging evidence suggests ARNI and SGLT2i may reduce arrhythmic events through favorable ventricular remodeling and electrophysiological effects, [10, 11, 12, 13, 14, 15, 16, 17, 18, 19, 20, 21, 22] raising the question of whether ICD benefit is attenuated in the modern era. Our findings, however, did not demonstrate a statistically significant reduction in ICD therapies in the contemporary‐era cohort. Importantly, these results should be interpreted as a lack of statistical significance rather than evidence of equivalence between eras.

The use of an era‐based classification was intended as a pragmatic approach to reflect the progressive implementation of contemporary GDMT in clinical practice. However, this strategy has inherent limitations. Despite significant differences between groups, a substantial proportion of patients in the contemporary‐era cohort were not treated with ARNI or SGLT2 inhibitors at baseline, while a small proportion of patients in the historical‐era cohort were already receiving these therapies. Therefore, treatment era does not fully capture individual exposure to GDMT, and residual heterogeneity in medical therapy must be acknowledged.

Furthermore, given the long duration of follow‐up, it is likely that a proportion of patients in the historical‐era cohort initiated ARNI and/or SGLT2 inhibitors during follow‐up, potentially attenuating differences between groups. This exposure crossover represents an important limitation and may have biased results toward the null.

A plausible explanation is that contemporary candidates for ICD implantation may represent a smaller, higher‐risk subgroup—those whose LVEF remains ≤ 35% despite optimal GDMT—thereby offsetting potential antiarrhythmic benefits of ARNI and SGLT2i. Additionally, being a real‐life cohort, this distribution reflects the reality of HFrEF medication throughout the duration of follow‐up, including the fact that ARNI is not well tolerated by all patients.

Subgroup analyses by ischemic and nonischemic etiology, as well as exploratory interaction testing in the full unmatched cohort, did not identify significant effect modifiers of the association between treatment era and ICD therapy, aside from a borderline signal for age older than 69 years. These findings were underpowered, hypothesis‐generating, and consistent with the main results.

This was an observational retrospective cohort study; therefore, no a priori sample size calculation was performed. However, a *post hoc* power analysis indicated that, with 234 matched patients, the study had 80% power to detect a hazard ratio of approximately 2.0 for the primary endpoint at a two‐sided α of 0.05. Accordingly, smaller but clinically meaningful differences between groups cannot be excluded, and the possibility of a Type II error should be considered when interpreting these results.

Importantly, the primary endpoint of this study, appropriate ICD therapy, reflects arrhythmic event burden rather than direct clinical benefit such as reduction in all‐cause mortality or sudden cardiac death. Although ICD therapies are clinically relevant, not all treated arrhythmias are life‐threatening, and therefore this endpoint may overestimate the true clinical benefit of ICD implantation. Consequently, our findings should not be interpreted as evidence that ICD benefit is maintained or unchanged in the contemporary era, but rather as a description of observed device therapy rates.

Cardiac MRI data, which could clarify the role of scar burden, were not systematically available and represent a limitation.

ICD programming in this study was relatively aggressive, with a VT detection threshold of 171 bpm and two ATP attempts before shock. This approach was consistent across both cohorts and was chosen to minimize prolonged arrhythmia episodes. While a higher detection threshold or more ATP attempts might reduce therapy incidence, such changes would have applied equally to both groups and are unlikely to explain the absence of a difference in outcomes.

Our results emphasize the need for adequately powered, prospective randomized trials to determine whether the prognostic benefit of ICD implantation for primary prevention persists in the current era of HFrEF management, and to clarify optimal patient selection and timing. This is in accordance with recent observational studies that have highlighted the need for a trial that can not only determine whether today ICD implantation today in primary prevention would still convey any prognostic benefit in patients with HFrEF (ICM and NICM) but also provide guidance on the timing of such intervention [23, 24, 25]. In the SAVE‐ICD [26] and DISCOVER‐ARNI [27] registries, a substantial proportion of patients no longer met ICD criteria after ARNI initiation.

Our findings align with recently published large‐scale data by Curtis et al., which analyzed contemporary ICD recipients, with nearly half of patients receiving appropriate ICD therapy by 10 years, with no evidence of declining therapy rates despite advances in heart failure care and device programming [28]. While that study provided a broad real‐world perspective, it did not capture patients' underlying medical therapy or distinguish ischemic from nonischemic etiologies. In contrast, our single‐center analysis specifically addressed the question of ICD utility in the setting of contemporary GDMT and used propensity‐score matching to balance baseline characteristics. Taken together, the two studies suggest that patients who remain candidates for primary‐prevention ICD implantation despite contemporary GDMT continue to experience clinically meaningful device‐treated arrhythmic events. Whether this reflects a persisting mortality benefit of ICD therapy in this higher‐risk subgroup cannot be determined from either study and warrants prospective evaluation.

Looking forward, the ongoing PROFID‐EHRA trial [29, 30]. will likely clarify ICD utility in the context of modern heart failure therapy and may influence future guidelines.

## 5 Limitations

The main limitation of our study is its retrospective and nonrandomized design. Although propensity‐score matching reduced baseline imbalances, residual confounding cannot be excluded.

The use of a temporal (era‐based) classification to define study groups represents an important limitation. While this approach was intended to reflect the progressive adoption of contemporary GDMT, treatment exposure remained heterogeneous. Some patients in the contemporary‐era cohort were not receiving ARNI or SGLT2 inhibitors at baseline, and a small proportion of patients in the historical‐era cohort were already taking these agents. Therefore, treatment era does not fully capture individual‐level exposure to GDMT.

In addition, given the long duration of follow‐up, it is likely that patients in the historical‐era cohort initiated ARNI and/or SGLT2 inhibitors during follow‐up, leading to exposure crossover between groups. This may have attenuated differences between cohorts and biased the results.

Granular, time‐varying data on GDMT initiation dates, titration, and discontinuation could not be reliably reconstructed. This is a single‐center cohort at a tertiary referral hospital spanning implantations from 2015 to 2022, including patients originally referred from other institutions. A substantial proportion of the historical‐era cohort has since died, and the national pharmacological prescription registry restricts record access after a patient's death; consequently, medication exposure could only be characterized as a binary status at the time of ICD implantation rather than as an individual‐level, time‐varying covariate.

The study was not designed or powered to assess equivalence between groups. Although no statistically significant difference was observed, the relatively small number of events and wide confidence intervals mean that clinically meaningful differences cannot be excluded, and a Type II error remains possible.

Standard errors for the primary Cox model were not clustered by matched pair. Fine‐Gray subdistribution hazard regression and inverse‐probability‐of‐censoring weighting were not available with our statistical software; we instead report cause‐specific Cox models and Aalen‐Johansen cumulative incidence functions to account for the competing risk of death. The expanded propensity‐score sensitivity analysis used complete‐case data (267/289 patients, 92.4%) and yielded fewer matched pairs (97 versus 117) due to reduced common support; although its primary result was consistent with the main analysis, residual imbalance persisted for several nontargeted covariates.

Another important limitation relates to the choice of primary endpoint. Appropriate ICD therapy reflects arrhythmic event burden but does not directly demonstrate clinical benefit, such as reduction in all‐cause mortality or sudden cardiac death. Furthermore, not all ICD therapies are necessarily life‐saving, which may lead to overestimation of clinical impact.

Additional factors that may influence ICD therapy rates were not fully captured. In particular, the distribution of device manufacturers was not systematically assessed, and differences in detection algorithms and therapy delivery across vendors may have influenced outcomes.

Cardiac MRI data, which could clarify the role of myocardial scar burden and arrhythmic substrate, were not systematically available and represent an additional limitation.

## 6 Conclusion

In this long‐term observational study, we did not observe a statistically significant difference in the incidence of appropriate ICD therapies between patients treated in a contemporary GDMT era and those in a historical‐era cohort. These findings should be interpreted cautiously, as the study was not powered to detect modest differences, and clinically meaningful effects cannot be excluded. Importantly, as the primary endpoint reflects device therapy rather than mortality or sudden cardiac death, our results do not establish whether the clinical benefit of ICD implantation is preserved or attenuated in the contemporary treatment era. Rather, our findings suggest that patients who continue to meet criteria for ICD implantation despite contemporary GDMT may represent a higher‐risk subgroup with persistent arrhythmic burden. Further adequately powered randomized studies are needed to define the role, timing, and patient selection for ICD implantation in the modern era of heart failure treatment.

## Funding

The authors have nothing to report.

## Conflicts of Interest

The authors declare no conflicts of interest.

## Supporting information


**Figure S1:** Formal treatment‐era × subgroup interaction tests (Cox models with era × subgroup interaction terms, full unmatched cohort, *n* = 289). Diamonds represent the interaction hazard ratio; horizontal lines represent 95% confidence intervals.
**Figure S2:** Covariate balance before and after propensity‐score matching, expressed as absolute standardized mean differences (SMD). Dashed reference line denotes the conventional SMD < 0.10 threshold for adequate balance.
**Table S1:** Expanded propensity‐score model (sensitivity analysis).
**Table S2:** Standardized mean differences, original versus expanded matching.

## Data Availability

The data that support the findings of this study are available on request from the corresponding author. The data are not publicly available due to privacy or ethical restrictions.
