# Supplementary material for: Appropriate ICD Therapies in Primary Prevention: A Propensity‐Matched Comparison of Contemporary and Historic Heart Failure Cohorts
Source: J Arrhythm. 2026 Oct 8;42(5):e70497. doi: 10.1002/joa3.70497 (PMC13650424; doi:10.1002/joa3.70497)
Supplement: Supplementary file 1 [file JOA3-42-e70497-s001.docx]

**Supplementary Material**

*Appropriate ICD Therapies in Primary Prevention: A Propensity-Matched Comparison of Contemporary and Historic Heart Failure Cohorts*

**Supplementary Figure 1**

*Supplementary Figure 1 - Formal treatment-era × subgroup interaction tests (Cox models with era × subgroup interaction terms, full unmatched cohort, n=289). Diamonds represent the interaction hazard ratio; horizontal lines represent 95% confidence intervals.*

**Supplementary Figure 2**

*Supplementary Figure 2 - Covariate balance before and after propensity-score matching, expressed as absolute standardized mean differences (SMD). Dashed reference line denotes the conventional SMD<0.10 threshold for adequate balance.*

**Supplementary Table 1 — Expanded propensity-score model (sensitivity analysis)**

Logistic regression model estimating the probability of contemporary-era cohort membership (complete-case analysis, n=267/289, 92.4%). Model c-statistic = 0.687.

| **Covariate** | **Coef.** | **SE** | **p** | **OR** | **95% CI** |
| --- | --- | --- | --- | --- | --- |
| Age (years) | 0.003 | 0.013 | 0.798 | 1.00 | 0.98-1.03 |
| Etiology (non-ischemic vs ischemic) | 0.048 | 0.294 | 0.870 | 1.05 | 0.59-1.87 |
| LVEF (%) | -0.030 | 0.025 | 0.222 | 0.97 | 0.92-1.02 |
| QRS ≥120ms | 0.905 | 0.307 | 0.003 | 2.47 | 1.35-4.52 |
| NYHA class (categorical) | 0.016 | 0.337 | 0.962 | 1.02 | 0.53-1.97 |
| Sex (male) | 0.006 | 0.342 | 0.987 | 1.01 | 0.51-1.97 |
| Hypertension | -0.634 | 0.332 | 0.056 | 0.53 | 0.28-1.02 |
| Diabetes mellitus type 2 | 0.844 | 0.311 | 0.007 | 2.33 | 1.26-4.28 |
| Dyslipidemia | -0.309 | 0.329 | 0.348 | 0.73 | 0.39-1.40 |
| Chronic kidney disease | -0.777 | 0.351 | 0.027 | 0.46 | 0.23-0.91 |
| COPD | 0.522 | 0.437 | 0.232 | 1.69 | 0.72-3.96 |
| Amiodarone use | 0.412 | 0.556 | 0.459 | 1.51 | 0.51-4.49 |

*Prior myocardial infarction and prior percutaneous coronary intervention were excluded from this model due to quasi-complete collinearity with etiology.*

**Supplementary Table 2 — Standardized mean differences, original *vs.* expanded matching**

| **Covariate** | **SMD pre-matching** | **SMD post (original model)** | **SMD post (expanded model)** |
| --- | --- | --- | --- |
| Hypertension | 0.325 | 0.318 | 0.090 |
| Diabetes type 2 | 0.198 | 0.268 | 0.000 |
| Dyslipidemia | 0.170 | 0.261 | 0.023 |
| COPD | 0.191 | 0.188 | 0.032 |
| Chronic kidney disease | 0.159 | 0.067 | 0.000 |
| Prior PCI | 0.403 | 0.397 | 0.329 |
| Prior MI | 0.246 | 0.210 | 0.190 |
| LVEF (%) | 0.204 | 0.264 | 0.129 |
| QRS ≥120ms | 0.313 | 0.058 | 0.116 |
| Age (years) | 0.062 | 0.004 | 0.096 |
| Male sex | 0.105 | 0.000 | 0.026 |
